# Supplementary material for: Novel Driver Strength Index highlights important cancer genes in TCGA PanCanAtlas patients
Source: PeerJ. 2022 Aug 11;10:e13860. doi: 10.7717/peerj.13860 (PMC9375969; doi:10.7717/peerj.13860)
Supplement: Supplemental Information 2 [file peerj-10-13860-s002.zip › COHORTS/genes plots/detailed/2021_8_16_14_9_distribution_events_detailed_males_10.pdf]

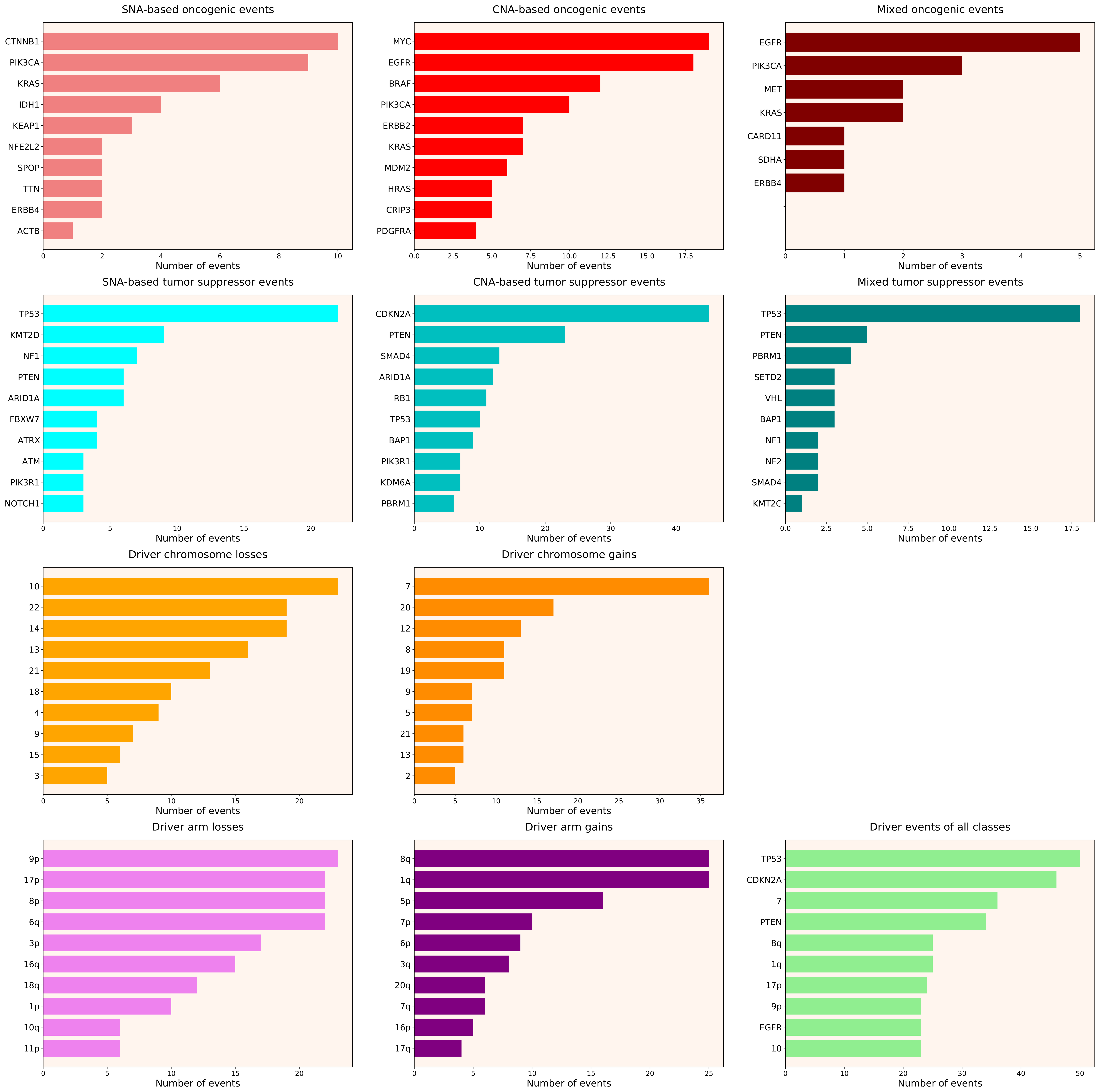
SNA-based oncogenic events
CTNNB1
PIK3CA
KRAS
IDH1
KEAP1
NFE2L2
SPOP
TTN
ERBB4
ACTB
0 2 4 6 8 10
Number of events
CNA-based oncogenic events
MYC
EGFR
BRAF
PIK3CA
ERBB2
KRAS
MDM2
HRAS
CRIP3
PDGFRA
0.0 2.5 5.0 7.5 10.0 12.5 15.0 17.5
Number of events
Mixed oncogenic events
EGFR
PIK3CA
MET
KRAS
CARD11
SDHA
ERBB4
0 1 2 3 4 5
Number of events
SNA-based tumor suppressor events
TP53
KMT2D
NF1
PTEN
ARID1A
FBXW7
ATRX
ATM
PIK3R1
NOTCH1
0 5 10 15 20
Number of events
CNA-based tumor suppressor events
CDKN2A
PTEN
SMAD4
ARID1A
RB1
TP53
BAP1
PIK3R1
KDM6A
PBRM1
0 10 20 30 40
Number of events
Mixed tumor suppressor events
TP53
PTEN
PBRM1
SETD2
VHL
BAP1
NF1
NF2
SMAD4
KMT2C
0.0 2.5 5.0 7.5 10.0 12.5 15.0 17.5
Number of events
Driver chromosome losses
10
22
14
13
21
18
4
9
15
3
0 5 10 15 20
Number of events
Driver chromosome gains
7
20
12
8
19
9
5
21
13
2
0 5 10 15 20 25 30 35
Number of events
Driver arm losses
9p
17p
8p
6q
3p
16q
18q
1p
10q
11p
0 5 10 15 20
Number of events
Driver arm gains
8q
1q
5p
7p
6p
3q
20q
7q
16p
17q
0 5 10 15 20 25
Number of events
Driver events of all classes
TP53
CDKN2A
7
PTEN
8q
1q
17p
9p
EGFR
10
0 10 20 30 40 50
Number of events
